# Point mutation bias in SARS-CoV-2 variants results in increased ability to stimulate inflammatory responses

**DOI:** 10.1038/s41598-020-74843-x

**Published:** 2020-10-20

**Authors:** Masato Kosuge, Emi Furusawa-Nishii, Koyu Ito, Yoshiro Saito, Kouetsu Ogasawara

**Affiliations:** 1 Department of Immunobiology, Institute of Development Aging and Cancer, 4-1 Seiryo-machi, Aoba-ku, Sendai, 980-8575 Japan; 2 Graduate School of Pharmaceutical Sciences, Tohoku University, Miyagi, Japan

**Keywords:** Immunology, Pathogenesis

## Abstract

Severe acute respiratory syndrome coronavirus 2 (SARS-CoV-2) infection induces severe pneumonia and is the cause of a worldwide pandemic. Coronaviruses, including SARS-CoV-2, have RNA proofreading enzymes in their genomes, resulting in fewer gene mutations than other RNA viruses. Nevertheless, variants of SARS-CoV-2 exist and may induce different symptoms; however, the factors and the impacts of these mutations are not well understood. We found that there is a bias to the mutations occurring in SARS-CoV-2 variants, with disproportionate mutation to uracil (U). These point mutations to U are mainly derived from cytosine (C), which is consistent with the substrate specificity of host RNA editing enzymes, APOBECs. We also found the point mutations which are consistent with other RNA editing enzymes, ADARs. For the C-to-U mutations, the context of the upstream uracil and downstream guanine from mutated position was found to be most prevalent. Further, the degree of increase of U in SARS-CoV-2 variants correlates with enhanced production of cytokines, such as TNF-α and IL-6, in cell lines when compared with stimulation by the ssRNA sequence of the isolated virus in Wuhan. Therefore, RNA editing is a factor for mutation bias in SARS-CoV-2 variants, which affects host inflammatory cytokines production.

## Introduction

Severe acute respiratory syndrome corona virus-2 (SARS-CoV-2) infection is the cause of a current worldwide pandemic first reported as a new type of pneumonia in Wuhan, China in December of 2019^1^. Since the first report from Wuhan, there is no complete convergence of virus infection in the world and SARS-CoV-2 infection causes high rate of morbidity and mortality^2^. In the prolonged SARS-CoV-2 infection, it is quite important to investigate whether the virulence of the virus have been changed.

Considered to have originated in bats with a possible unknown intermediate reservoir host^3^, SARS-CoV-2 has a nucleotide sequence close to that of SARS-CoV, a virus causing severe pneumonia^4^ that was the cause of a human outbreak in 2003. SARS infections induce an excessive immune response called a cytokine storm, which causes severe pneumonia. It has been reported that the production of TNF-α and IL-6 is increased in proportion to the severity of symptoms in SARS-CoV-2 patients^5^, and it is considered that cytokine storm is a factor of severe pneumonia^6,7^. Unexpectedly, minimal amounts of type I IFNs have been detected in the peripheral blood or lungs of patients with severe SARS-CoV-2 infection^8^.

In virus-infected hosts, inflammatory cytokines such as TNF-α and IL-6 are produced via the pattern recognition receptors (TLRs)^9^. RNA viruses are recognized via TLR7 and TLR8^10^. Among RNA sequences, TLR7 and TLR8 recognize the specific pattern of the nucleotide sequence of the virus. TLR7 preferentially recognizes the continuous U, and TLR8 prefers GU sequences^11–13^. Thus, it might be possible to speculate the ability of cytokine production from the RNA sequences of virus.

It is thought that coronaviruses have fewer gene mutations than other RNA viruses due to the presence of an RNA-proofreading enzyme in their genomes. In classic SARS-CoV, the non-structural protein 14 (nsp14) gene encoded by open reading frame (ORF) 1b has 3′ to 5′ exoribonuclease activity typical of RNA-proofreading enzymes^14,15^. Nevertheless, according to previous genomic analysis, SARS-CoV-2 has several variants classified into three types, A, B, and C^16^. Although these SARS-CoV-2 variants contain several gene mutations, the influence of these mutations on infection are unknown.

Cell-derived RNA editing enzymes are a factor to induce point mutation in viral genome including RNA viruses^17–19^. RNA editing enzymes have substrate specificity and context preferences around target sites^20–22^. RNA editing enzymes such as adenosine deaminases acting on RNAs (ADARs) and apolipoprotein B mRNA editing-enzyme, catalytic polypeptides (APOBECs) have been studied in RNA virus infections. ADARs are enzymes that extract an amino group from adenosine and convert it into inosine, a function mainly exerted on double-stranded RNA^22^. APOBECs, a family of cytidine deaminases, are enzymes that extract an amino group from cytidine and convert it into uracil (U)^20^. APOBECs have been reported to function on ssDNA as a substrate^20^. Furthermore, APOBEC1, APOBEC3A and APOBEC3G, also recognize single strand RNA (ssRNA) as a substrate^23–25^. Interestingly, involvement of APOBEC3 has been suggested in mutation of the coronavirus NL63^26^. However, it is unknown whether mutations of SARS-CoV-2 variants are induced by host RNA editing. To this end, we investigated SARS-CoV-2 mutations to determine the likelihood they were caused by host RNA editing, and assessed the effects of these mutations on host cell cytokine production.

## Materials and methods

### Acquirement of sequence data

SARS-CoV-2 sequences (8,845) were downloaded from the Global initiative on sharing all influenza data (GISAID) database (https://www.gisaid.org/). They were available as of May 20, 2020, collected by March 23, 2020, and annotated as “complete (> 29,000 bp)”, “high coverage”, and “low coverage excl”, “Human” in host. These sequences were provided by 342 laboratories, a list of which is shown in Supplementary Table S1 and S2. In addition, sequences with the following comments from GISAID were excluded: (1) N > 0.50%, (2) frame shift, (3) unique mutation > 0.10%, (4) low coverage, and (5) ambiguous collection date. Also, sequences with duplicate names and mismatches in alignment and without correct collection dates were excluded. This resulted in 7804 sequences for analysis.

### Phylogenetic network analysis

To build a phylogenetic tree of the SARS-CoV-2 sequence, Augur pipeline published by Nextstrain (github.com/nextstrain/ncov) was used^27,28^. The first reported genome in Wuhan (MN908947) was used as the reference genome for the alignment. We also masked 130 bases from the beginning and 100 bases from the end after alignment. The calculated phylogenetic tree was then used for analysis. The phylogenetic tree was visualized using Auspice.

### Sequence context analysis

“Context” represents the upstream (negative number) and downstream (positive number) sequence of target sites. These contexts were taken from unmasked region of the reference genome (MN908947).

We defined “Observed proportion” and “Expected proportion” as follows, and calculated the respective values.

“Observed proportion” represents the proportion that the specific base exists at position + 1 (or −1) of mutated site.

Observed proportion (%) = total counts of position −1 (or + 1) residue at mutated base/total numbers of each mutated base × 100.

“Expected proportion” represents the proportion that specific base exists at position −1 (or + 1) of A, (U, G or C) in unmasked region of reference genome.

Expected proportion (%) = total counts of position −1 (or + 1) residue / total numbers of each base × 100.

To analyze the features of contexts, we compared the observed proportion to the expected proportion.

In Fig. 3E,F, we set context on − 3 ~  + 3, and performed the above calculation. The counts used to calculate the observed and expected proportions in Fig. 3A–F were shown in Supplementary Table S3 and S4.

### Single strand RNA sequence used for cell stimulation

For the cell stimulation assay, four different sequences, EPI_ISL_419308, EPI_ISL_415644, EPI_ISL_418420, and EPI_ISL_419846, were selected from SARS-CoV-2 variants. These mutated sequences were detected in Japan, Georgia, France and Australia, respectively. Within the full length of ssRNA of each of the four variants, one region where the mutation to U was observed was extracted and synthesized. The ssRNA sequences derived from these different variants were as follows: variant-1 (5′-AUUUAUUGUUCUUUUUACCC-3′; at 2946–2965 region in EPI_ISL_419308), variant-2 (5′-UUUUUGUUCUUUUUUUUUUA-3′; at 11,041–11,060 region in EPI_ISL_415644), variant-3 (5′-UUUCUACAGUGUUCCCACUU-3′; at 14,392–14,411 region in EPI_ISL_418420), and variant-4 (5′-AAACCUUUUGAGAGAGUUUU-3′; at 22,946–22,965 in EPI_ISL_419846). As the control for mutated SARS-CoV-2 sequences, the same regions from the reference sequence (MN908947) was used. The reference sequences corresponding to each four different mutant were as follows; Wuhan-1 (5′-AUGUAUUGUUCUUUCUACCC-3′; at 3023–3042 region), Wuhan-2 (5′-UCUUUGUUCUUUUUUUUGUA-3′; at 11,066–11,085 region), Wuhan-3 (5′-UCUCUACAGUGUUCCCACUU-3′; at 14,390–14,409 region), and Wuhan-4 (5′-AAACCUUUUGAGAGAGAUAU-3′; at 22,946–22,965 region). A sequence not containing U (5′-GACAGAGAGAGAACAAG-3′) was used as a negative control for induction of TLR7 mediated cytokine production. ssRNAs were synthesized by Nihon Gene Research Laboratories. Inc. (Sendai, Miyagi, Japan).

### Cell culture and stimulation with ssRNA

The human monocytic leukemia cell line, THP-1, was maintained in RPMI-1640 medium supplemented with 10% FCS, 55 mM 2-mercaptoethanol, 100 mM non-essential amino acids (NEAAs), 1 mM pyruvic acid and 20 mM ml^−1^ each penicillin and streptomycin. 4 × 10^5^ cells were cultured in 150 μl RPMI using a 96 well flat bottom plate.

We performed pseudo-infection model following Yan Li et al.^29^. For the measurement of human TNF-α, cells were cultured in the presence of PMA (0.2 ng/ml, Sigma Aldrich, St. Louis, MO, USA) and stimulated by 160 pmol ssRNA with DOTAP (10 μg, Roche Diagnostics, Mannheim, Germany). For measurement of human IL-6, cells were cultured in the presence of PMA (50 ng/ml) and stimulated using 480 pmol ssRNA with DOTAP (15 μg).

### Detection of cytokines

Human TNF-α and IL-6 were measured in culture supernatants using OptEIA sets (BD Bioscience, San Diego, CA USA). Culture supernatant was collected at 18 h for the detection of TNF-α, and at 48 h for IL-6.

### Statistical analyses

Binomial test was performed using scipy 1.4.1 of the Python 3 base package. Mann–Whitney U test was performed using Prism 8 software (GraphPad Software, San Diego, CA).

## Results

### Point mutations in SARS-CoV-2 variants

**Figure 1:**
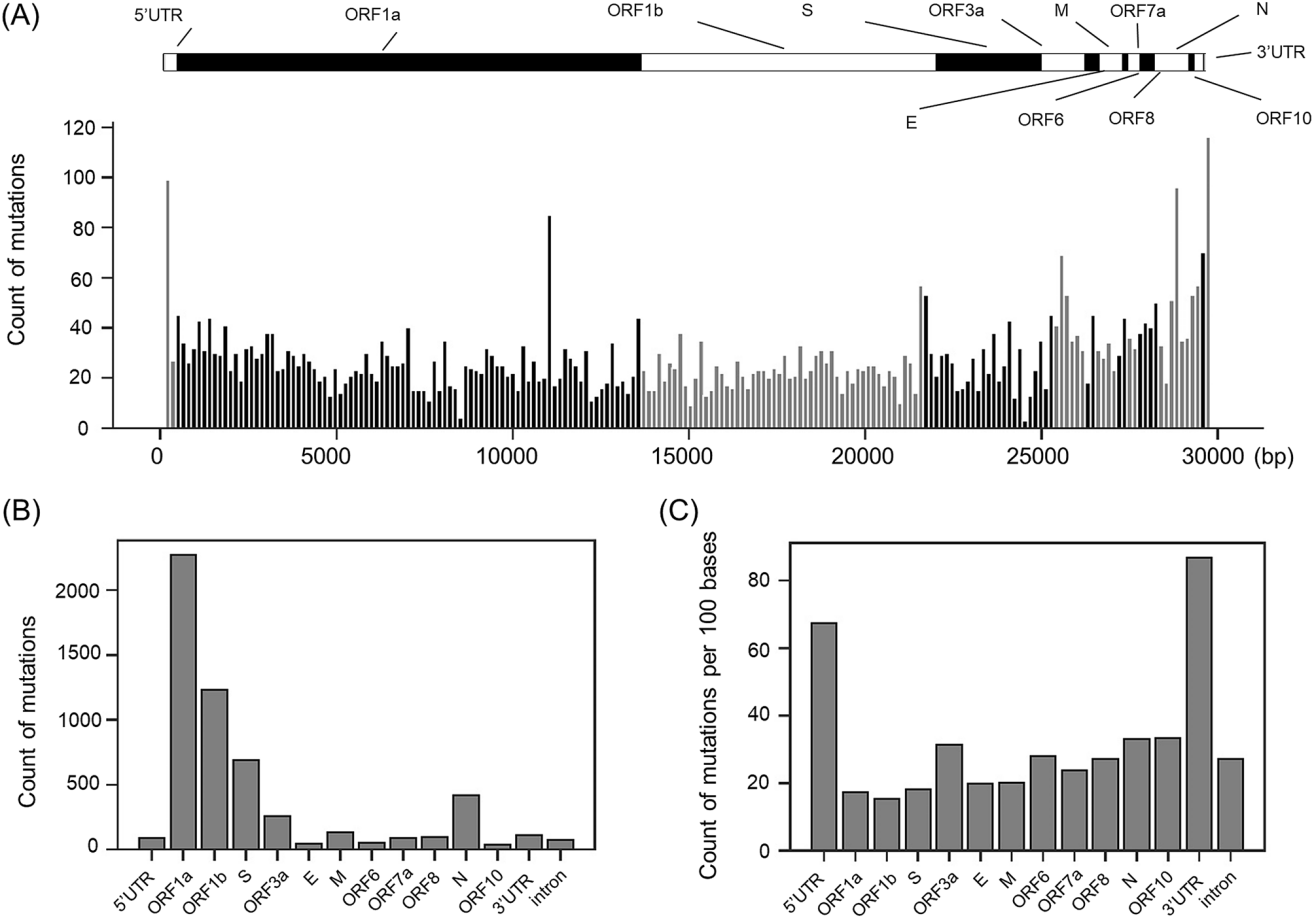
Distribution of point mutations in the SARS-CoV-2 genome. (**A**) Upper illustration represents the location of each gene in the full-length single strand RNA (ssRNA). The lower histogram shows the numbers of mutations at each location. Each bar represents SARS-CoV-2 variants grouped in bins of 150 nucleotides. (**B**) Number of point mutations per genome. (**C**) Number of point mutations in each gene divided by gene length.

To investigate the frequency of point mutations in SARS-CoV-2 variants, we performed phylogenetic network analysis using the 7,804 sequences published in GISAID. These sequences were collected until March 23, 2020 and over 5000 times of point mutation were calculated by the phylogenetic network analysis. Next, we analyzed the locations of these point mutations (Fig. 1A). Although the average number of point mutations per 150 nucleotides (bin) was about 28, we observed a higher frequency of point mutations in several locations. To further analyze the polarization of point mutations in each gene, we counted the number of point mutations per gene. As shown in Fig. 1B, there were more point mutations in ORF-1a and ORF-1b. However, as shown in Fig. 1A, open reading frame (ORF)-1a and ORF-1b are much longer than other regions, which may result in more mutations; hence, we estimated the rate of point mutations per 100 bases in each gene (Fig. 1C). When normalized by gene length, the highest frequency of point mutations occurred in the 5′-untranslated region (UTR) and 3′-UTR. These results indicate that point mutations are present in SARS-CoV-2 variants; however, they do not cluster within the gene coding regions.

### Point mutations in SARS-CoV-2 variants are biased with disproportionate mutation to U

**Figure 2:**
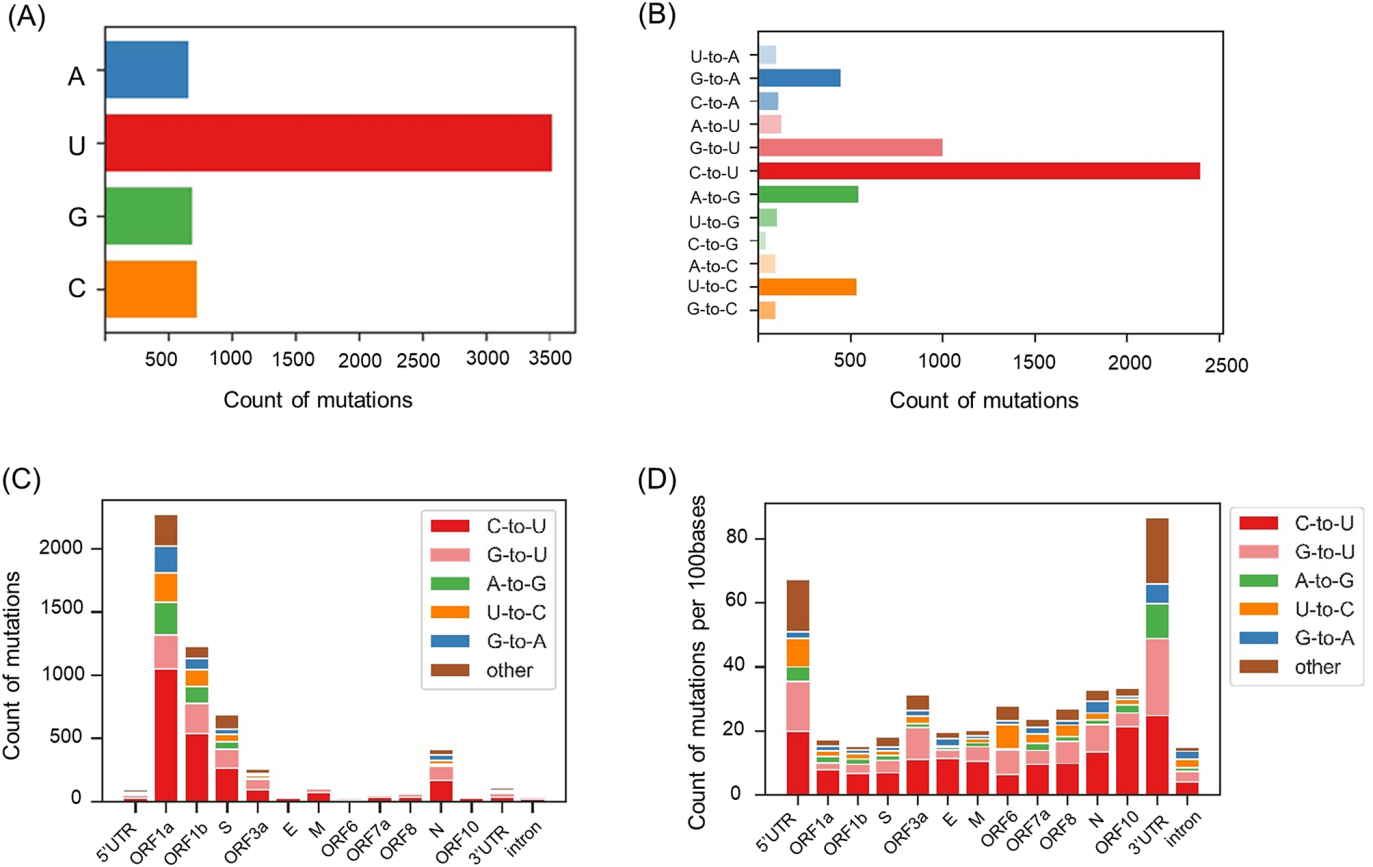
The bias of point mutations in SARS-CoV-2 variants. (**A**, **B**) The number of point mutations in SARS-CoV-2 variants. The Y axis of (**A**) represents the substituted base after the point mutation, and the Y axis of (**B**) shows the original base and substituted base at each point mutation. (**C**) The mutation patterns in each gene. (**D**) Number of point mutations in each gene divided by gene length.

Next, we focused on the mutated bases and examined the features of point mutations in SARS-CoV-2 variants. Analysis of the frequency of the substituted base after the point mutation revealed that mutation to Uracil (U) occurred approximately four times more often than did Adenine (A), Cytosine (C), or Guanine (G) (Fig. 2A). Further analysis revealed that the point mutation to U was mainly derived from C (around 2400 total mutations) or G (around 1000 total mutations), but rarely from A (around 100 mutations) (Fig. 2B). Moreover, point mutation from G to A (G-to-A), A-to-G, and U-to-C were also prominent, occurring about 500 times each. This bias in mutations suggests the involvement of host RNA editing since it is known that APOBECs can cause C-to-U and G-to-A mutations, while ADARs cause A-to-G and U-to-C mutations. Interestingly, the results in Fig. 2B are partially consistent with the known substrate specificity of APOBECs and/or ADARs. We further analyzed the mutation bias per gene, and found that the mutation pattern was similar between genes (Fig. 2C,D). These results indicate that point mutations in SARS-CoV-2 variants are significantly biased with disproportionate mutation to U. The mutation patterns were partially consistent with the APOBECs-induced and ADARs-induced point mutations, suggesting the involvement of RNA editing enzymes.

### Context preferences at the mutation site in SARS-CoV-2 variants

**Figure 3:**
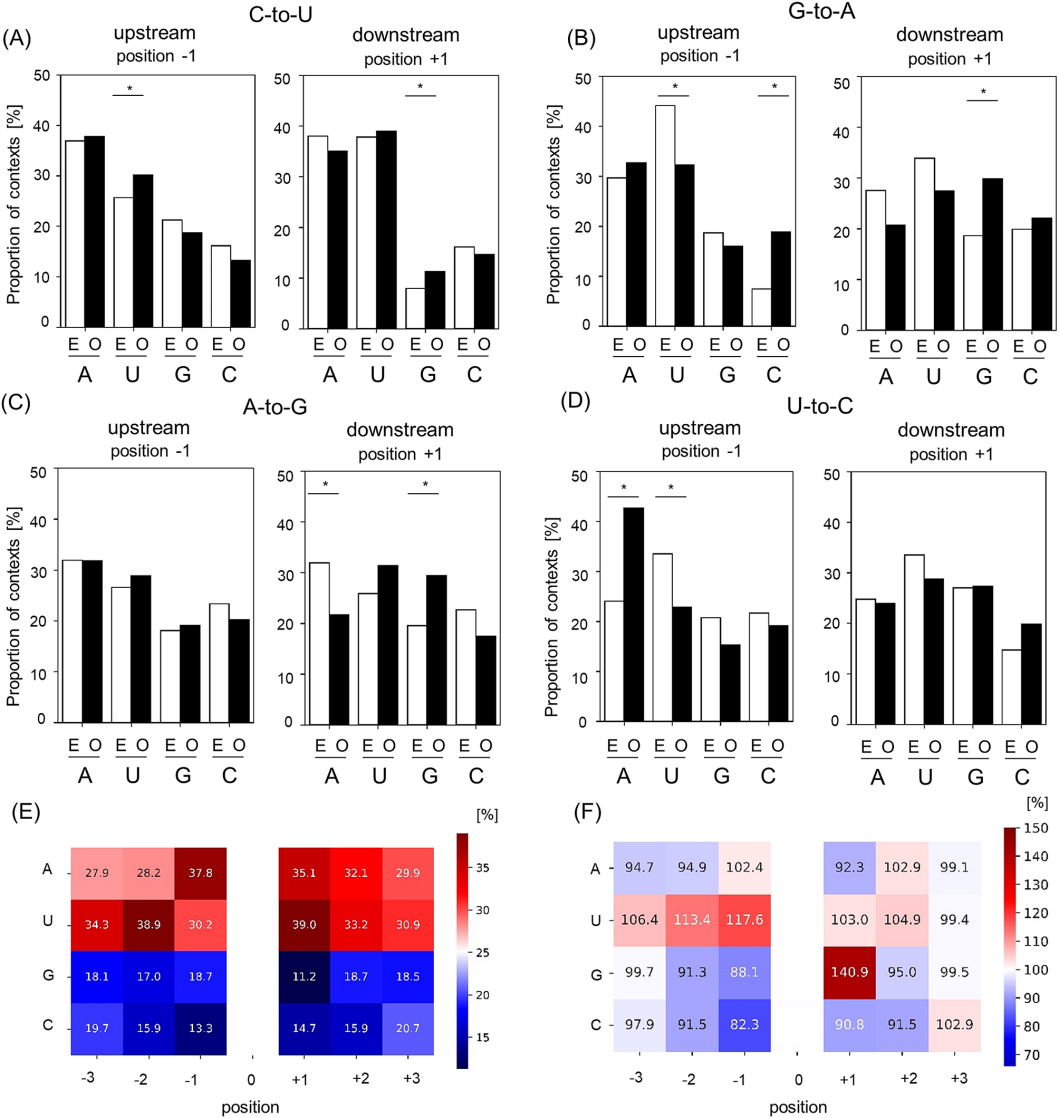
The details of contexts at mutated site. The context of a base each upstream (− 1) and downstream (+ 1) of following mutations were shown respectively, C-to-U (**A**). G-to-A (**B**). A-to-G (**C**). U-to-C (**D**). Figures on the left depicts a base of upstream (−1) of the mutated sites and on the right depicts a base of downstream (+ 1) of the mutated sites. White bars represent the expected proportion (**E**) and black bars represent the observed proportion (O). *p < 0.00001. (**E**) The observed proportion of contexts at three bases each upstream and downstream of C-to-U mutations was shown. The 0 indicates the mutation site and negative and positive numbers indicate sites upstream and downstream, respectively. (**F**) The Ratio [%] of observed proportion to expected proportion in the context of C-to-U. The observed proportion and expected proportion were calculated as shown in materials and methods.

The above results indicate the involvement of host RNA editing machinery. Since “context preferences” support the involvement of RNA editing machinery, we set the “Contexts” which represents the upstream and downstream sequence of mutated site, and analyzed the context preferences. C-to-U and G-to-A mutations are consistent with those caused by APOBECs, and A-to-G and U-to-C are characteristic of ADARs. To examine the involvement of these two kinds of enzymes, we chose four patterns (C-to-U, G-to-A, A-to-G, and U-to-C) and analyzed the details of contexts which are adjacent to one base upstream (−1) and downstream (+ 1). In C-to-U mutation, the observed proportion of U at position −1 and G at position + 1 was markedly increased as compared with their expected proportion respectively (Fig. 3A). In G-to-A mutation, C at position −1 and G at position + 1 was increased. Conversely, U at the position + 1 was decreased (Fig. 3B). Similarly, at position + 1 in A-to-G mutation, G was increased but A was decreased (Fig. 3C). At position −1 in U-to-C mutation, A was increased but U was decreased (Fig. 3D). These biases in the contexts indicate that specific base is preferred at position + 1 or −1 in every 4 patterns of point mutations, suggesting the context preference.

Moreover, our results reflected the context preferences of APOBECs and ADARs. The increase of U at position −1 is consistent with the involvement APOBEC3s (Fig. 3A). The increases of G at position + 1 in G-to-A mutation site suggest the involvement of APOBEC3G (Fig. 3B). Because APOBEC3G prefers C at position −1 of C-to-U mutations, in other words, when SARS-CoV-2 is replicated, APOBEC3G leads to C-to-U mutations on complementary RNA, resulting in the induction of G-to-A mutation with G at its position + 1 in viral genome. The increase of G at position + 1 in A-to-G mutation site is consistent of the context preferences of ADARs (Fig. 3C).

In most commonly observed point mutation, C-to-U, we expanded contexts which are the three bases upstream (−3) and downstream (+ 3) of mutated site (Fig. 3E, F). Although we found a high abundance of A and U in the observation proportion (Fig. 3E), the SARS-CoV-2 genome contains a high proportion of A and U residues (A: 30%, U: 32%). To exclude the AU-rich bias in SARS-CoV-2 sequence, we calculated the ratio of observed proportion to expected proportion (Fig. 3F). We found that U was more frequently present at position −1 and position −2 (*p* < 0.00001). This is consistent with the sequence specificity of APOBEC3s. In addition, G was more commonly found at position + 1 (*p* < 0.00001).

These context preferences provide evidence that the RNA editing machinery contributes to the induction of point mutations. Moreover, APOBECs and ADARs are the strong candidate to induce point mutations in SARS-CoV-2 variants.

### SARS-CoV-2 variants with increased prevalence of U induce augmented production of inflammatory cytokines

**Figure 4:**
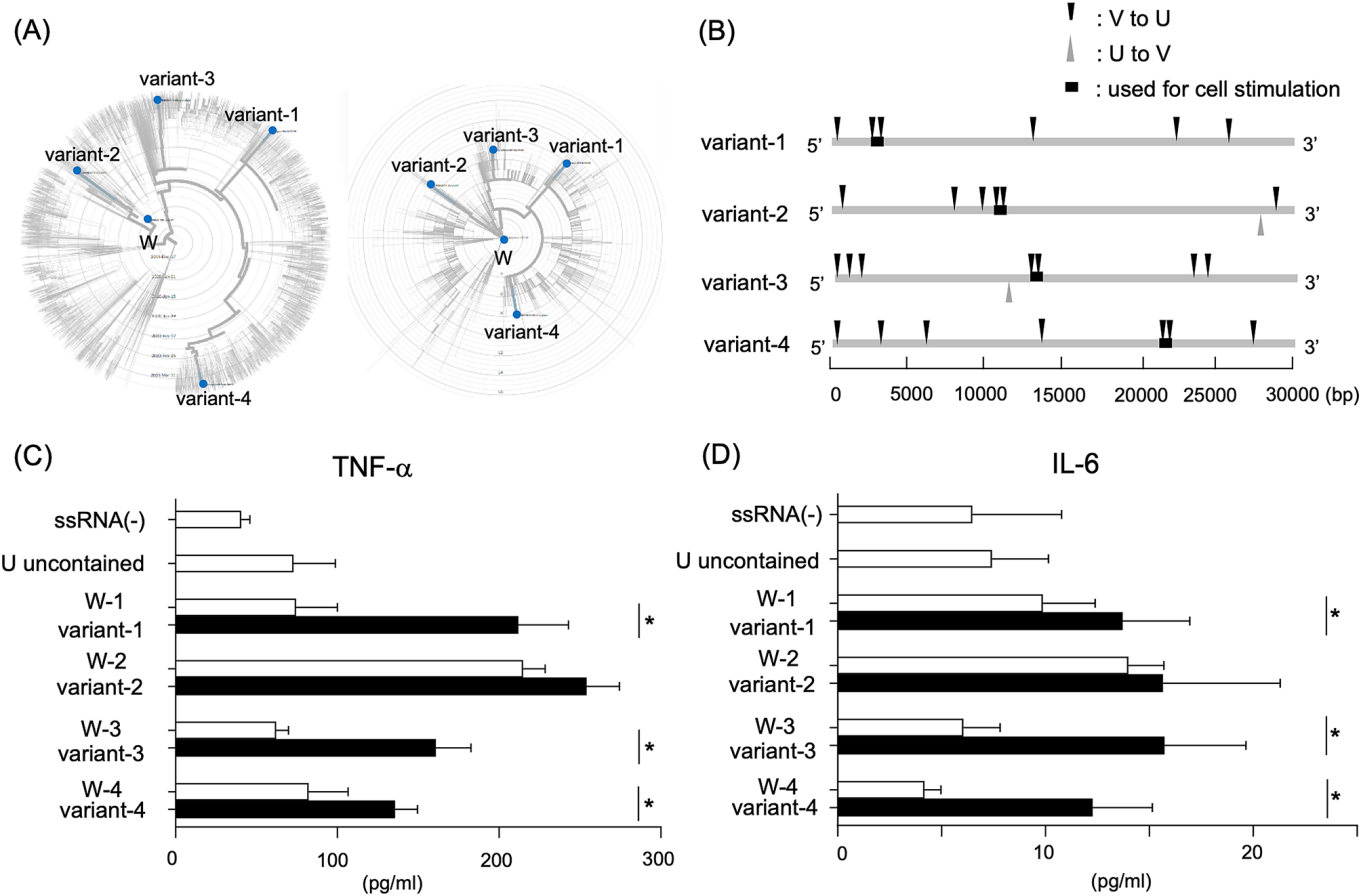
Increased proportion of U in the genome caused by point mutations enhances inflammatory cytokine production. (**A**) Phylogeny of four SARS-CoV-2 variants. The distance from the center represents time (left) and diversity (right) from ancestor, respectively. W indicates the original SARS-CoV-2 sequence reported in Wuhan (MN908947). (**B**) The location of mutations to U or from U in four different SARS-CoV-2 variants. Downward triangle shows the mutation from V to U, and upward triangle shows the mutation from U to V, where V represents all bases other than U. Square symbol shows the ssRNA sequences used for cell stimulation. (**C**, **D**) ssRNA-induced TNF-α and IL-6 production. THP-1 cells were stimulated with 160 pg ssRNA with 10 μg DOTAP for detection of TNF-α, and 480 pg ssRNA with 15 μg DOTAP for detection of IL-6. The production of TNF-α was measured after 18 h stimulation (**C**), and the production of IL-6 was measured after 48 h stimulation (**D**). Values are means ± SD (n = 6). Data are representative of two independent experiments with similar results. *p < 0.05.

To examine the frequency of point mutations to U within the full length of each RNA sequence, we picked four different sequences from SARS-CoV-2 variants (Fig. 4A). These four different sequences were derived from Japan, Georgia, France, and Australia. As shown in Fig. 4B, the frequency of point mutations to U was much higher than the frequency of U to A, G, or C. Previously, several studies showed that U-rich ssRNA stimulates innate immune cells through TLR7 signaling to produce inflammatory cytokines^9,10^. Thus, we hypothesized that the large number of U residues resulting from point mutations enhances the induction of inflammatory cytokines by human macrophages. To this end, we analyzed the production of TNF-α and IL-6 in the human monocyte/macrophage cell line, THP-1, stimulated by U-rich region of SARS-CoV-2 variants (Fig. 4B, square symbol). As expected, ssRNA sequences lacking U residues did not upregulate the production of TNF-α (Fig. 4C). The increment of U numbers induced by point mutation enhanced the cytokine productions in variant-1, 3 and 4, comparing with the stimulation by reference ssRNA sequence from Wuhan. The production of IL-6 was lower than TNF-α, however, we observed the similar tendency in the production of IL-6 (Fig. 4D). Of course, we also observed that mRNA expression of TNF-α and IL-6 are induced by ssRNA stimulation of variant-1, 3 and 4, compared with the stimulation of Wuhan ssRNA (Supplemental Fig. 1). These results demonstrate that point mutation to U within the SARS-CoV-2 genome results in the ability to stimulate increased production of inflammatory cytokines such as TNF-α and IL-6.

## Discussion

In this study, we found that point mutations in SARS-CoV-2 variants are significantly biased with disproportionate mutation to U. The tendency of mutation biases and context preferences suggest the contributions of two kinds of RNA editing enzymes, APOBECs and ADARs, as inducers of point mutations in SARS-CoV-2 variants.

Furthermore, our data suggest that the increase in U residues in the SARS-CoV-2 genome enhance inflammatory cytokine production by innate immune cells. Specifically, we show that U-rich ssRNA sequences from SARS-CoV-2 variants upregulate TNF-α and IL-6 production by a human monocyte/macrophage cell line. The increase of U residues due to viral mutations resulted in the production of inflammatory cytokines from macrophages. This is presumed to activate the innate immune system. Since the ability to eliminate the virus is enhanced by the activation of innate immunity, as a result, the toxicity of SARS-CoV-2 may be attenuated by the mutation. In addition, since minimal amount of type I IFNs was detected from SARS-CoV-2 patients, we focused on proinflammatory cytokines such as TNF-α and IL-6. Further studies are required to examine the molecular mechanisms underlying the disproportionate production of cytokines.

In the series of sequences in SARS-CoV-2 variants, we found the gene cording region of RNA-proofreading enzymes (nsp14). Studies by Shannon et al. have also shown that the sequence of nsp14 in SARS-CoV-2 variants is very similar to that in classic SARS-CoV and the motif of nsp14 is conserved in SARS-CoV-2 variants^30^. This suggests nsp14 may be responsible for the lower mutation rate of SARS-CoV-2 variants compared to other RNA viruses. Given that the error correcting ability of nsp14 decreases the frequency of mutations in the viral genome, the fact that there is a bias in mutations seen in SARS-CoV-2 variants is striking.

It is known that several adenine and cytosine are spontaneously deaminated into inosine and uracil, respectively. However, by the following two reasons from our results, the point mutations are induced not at random but through biochemical machinery. One is that the point mutations were significantly biased. The other is that the preferred contexts are present at the mutated sites.

RNA editing enzymes a factor which induces biased point mutations. The four mutation patterns (C-to-U, G-to-A, A-to-G, U-to-C) were frequently detected in SARS-CoV-2 variants. Interestingly, these four patterns were consistent with the substrate specificity of APOBECs and ADARs. APOBECs induce C-to-U and G-to-A mutations. ADARs induce A-to-G and U-to-C mutations. Moreover, we found the specific sequences around mutation sites, which match the context preferences of APOBECs and ADARs. These results suggest that APOBECs and ADARs are the strong candidate to induce point mutations in SARS-CoV-2 variants. The recent studies by Di Giorgio et al. also suggest that APOBECs and ADARs are involved in point mutations of SARS-CoV-2^31^ by analyzing RNA sequences from bronchoalveolar lavage fluids obtained from COVID-19 patients. In addition, as supportive data, some APOBEC3s or ADARs are upregulated in lung epithelial cell lines; Calu-3 by infection of SARS-CoV-2 according to The Immunological Genome Project (ImmGen)^32,33^.

In SARS-CoV-2 variants, our results suggest that APOBECs rather than ADARs more contribute to induction of point mutations. Because C-to-U mutations were the most frequent, and their context preferences were consistent with APOBECs. As supportive data, compared to healthy lung biopsies, RNA editing enzymes tend to be upregulated in SARS-CoV-2 patient lung biopsies. Especially, increase rate of APOBEC3A is higher than that of ADARs according to ImmGen. The human genomes contain several *apobec* genes, including APOBEC1, APOBEC2, AID, and APOBEC3A-APOBEC3H. APOBEC3F has been reported to contribute to Human Immunodeficiency virus (HIV)-1^34^, and APOBEC3G has been reported to mutate hepatitis B virus (HBV)^35^. Recently several studies have shown that the APOBECs also interact with ssRNA of RNA viruses. As shown in Fig. 3A,F, our results indicate the contribution of APOBEC3s for the mutation of C-to-U in SARS-CoV-2 variants due to detection of U at position −1 and position −2 of the C-to-U mutation sites. It should be noted that we also observed a high frequency of G-to-U mutations in SARS-CoV-2, which is not likely an effect of APOBECs or ADARs. It has been reported that 8-oxoguanine is one of the candidate molecules for causing G-to-T mutation in mouse DNA^36^. Although it cannot deny that 8-oxoguanine may also be involved in G-to-U mutation of SARS-CoV-2, we do not have further information of G-to-U mutations in SARS-CoV-2. Therefore, further studies are needed to identify other biochemical mechanisms involved in this process.

For TLRs that recognize RNA sequences, it has been reported that increased levels of U enhance the reactivity of TLR7^10^. Consistent with previous report, the cytokine production from THP-1 stimulated by ssRNA were dependent on TLR7 (Supplemental Fig. 1). As shown in Fig. 4C, D, increased U frequency in the SARS-CoV-2 genome enhances TNF-α and IL-6 production in our pseudo-infection model. Our phylogenetic network analysis using 7804 sequences of SARS-CoV-2 variants reveals that the mutations to U were over 2500-times more frequent than mutations from U. In addition, as shown in Fig. 4B, the number of Us within the full length ssRNA in each SARS-CoV-2 variant is significantly increased compared to the original isolate. Thus, the ability of full-length mutated ssRNA to induce inflammatory cytokines may be greater than that of the original isolate. These results suggest that this could be one mechanism via which SARS-CoV-2 gene mutations cause increased inflammatory activation.

Our findings suggest that the biased point mutations of SARS-CoV-2 variants are induced by RNA editing during the host defense reaction against viruses. Thus, the evolution of this virus results in increased immune activation due to the selective pressure of host defense.

## Supplementary information

Supplementary file1

Supplementary file2

Supplementary file3

Supplementary file4

Supplementary file5

Supplementary file6
