# Supplementary material for: Point mutation bias in SARS-CoV-2 variants results in increased ability to stimulate inflammatory responses
Source: Sci Rep. 2020 Oct 20;10:17766. doi: 10.1038/s41598-020-74843-x (PMC7575582; doi:10.1038/s41598-020-74843-x)
Supplement: Supplementary file 1 — Supplementary file1 [file 41598_2020_74843_MOESM1_ESM.docx]

**Supporting information**

**Point mutation bias in SARS-CoV-2 variants results in increased ability to stimulate inflammatory responses**

Masato Kosuge^1,2#^, Emi Furusawa-Nishii^1#^, Koyu Ito^1^, Yoshiro Saito^2^,

Kouetsu Ogasawara^1*^

^1^ Department of Immunobiology, Institute of Development Aging and Cancer, and ^2^Graduate school of Pharmaceutical Sciences, Tohoku University, Miyagi, Japan

#The two authors equally contributed.

***Correspondence:*** Kouetsu Ogasawara, Department of Immunobiology, Institute of Development Aging and Cancer, 4-1 Seiryo-machi, Aoba-ku, Sendai 980-8575, Japan. *E-mail*; immunobiology@grp.tohoku.ac.jp Fax/telephone: +81-022-717-8452/ +81-022-717-8579

**Supplementary Materials and Methods**

*RNA interference and cell stimulation*

For TLR7 silencing studies, 30nM of negative control or human TLR7 Silencer small interfering RNA (siRNA) (AM4613, AM16708, Thermo Fisher Scientific) was mixed with Lipofectamine 3000 (Invitrogen, Carlsbad, CA) following the manufacturer’s instructions, and added onto the 4 x10^5^ of THP-1 cells. 18 hours after the transfection, cell stimulation was performed. 320pmol of ssRNA was mixed with DOTAP Liposomal Transfection Reagent (10μl) following the manufacturer’s instructions, and added onto the siRNA-transfected THP-1 cells under the presence of PMA. The concentration of PMA was 1ng/ml for TNF-α mRNA detection and 5ng/ml for IL-6 mRNA detection. Cells were harvested at the indicated time points.

*Quantitative RT-PCR*

Total RNA was extracted from siRNA-transfected and ssRNA-stimulated THP-1 cells using RNeasy Mini Kit (QIAGEN, Hilden, Germany). Total RNA was reverse transcribed using SuperScript III with Oligo (dT) as the primer (Invitrogen, Carlsbad, CA). Quantitative RT-PCR was performed using the QuantStudio 3 (Applied Biosystems, Foster City, CA) and *Power* SYBR Green PCR Master Mix (Thermo Fisher Scientific, Waltham, MA) according to the manufacturer’s instructions. Specific RNA primers were as follows; human GAPDH: forward 5’-GTCTCCTCTGACTTCAACAGCG-3’, reverse5’-ACCACCCTGTTGCTGTAGCCAA-3’, human TNF-α: forward 5’-CTCTTCTGCCTGCTGCACTTTG-3’, reverse 5’-ATGGGCTACAGGCTTGTCACTC-3’, human IL-6: forward 5’-AGACAGCCACTCACCTCTTCAG-3’, reverse 5’-TTCTGCCAGTGCCTCTTTGCTG-3’. Data were presented as the ratio to GAPDH.

Supplementary figure 1: mRNA expression of TNF-α and IL-6 by siRNA-transfected and ssRNA-stimulated THP-1 cells

siRNA-transfected and ssRNA-stimulated THP-1 cells were subjected to quantitative RT-PCR. Cells were harvested at one hour for TNF-α mRNA analysis (A), and at 6 hours for IL-6 mRNA analysis (B). Values are means±SD (n=3 to 6) **p*<0.05.
