# Supplementary figures and images for: Point mutation bias in SARS-CoV-2 variants results in increased ability to stimulate inflammatory responses
Source: Sci Rep. 2020 Oct 20;10:17766. doi: 10.1038/s41598-020-74843-x (PMC7575582; doi:10.1038/s41598-020-74843-x)

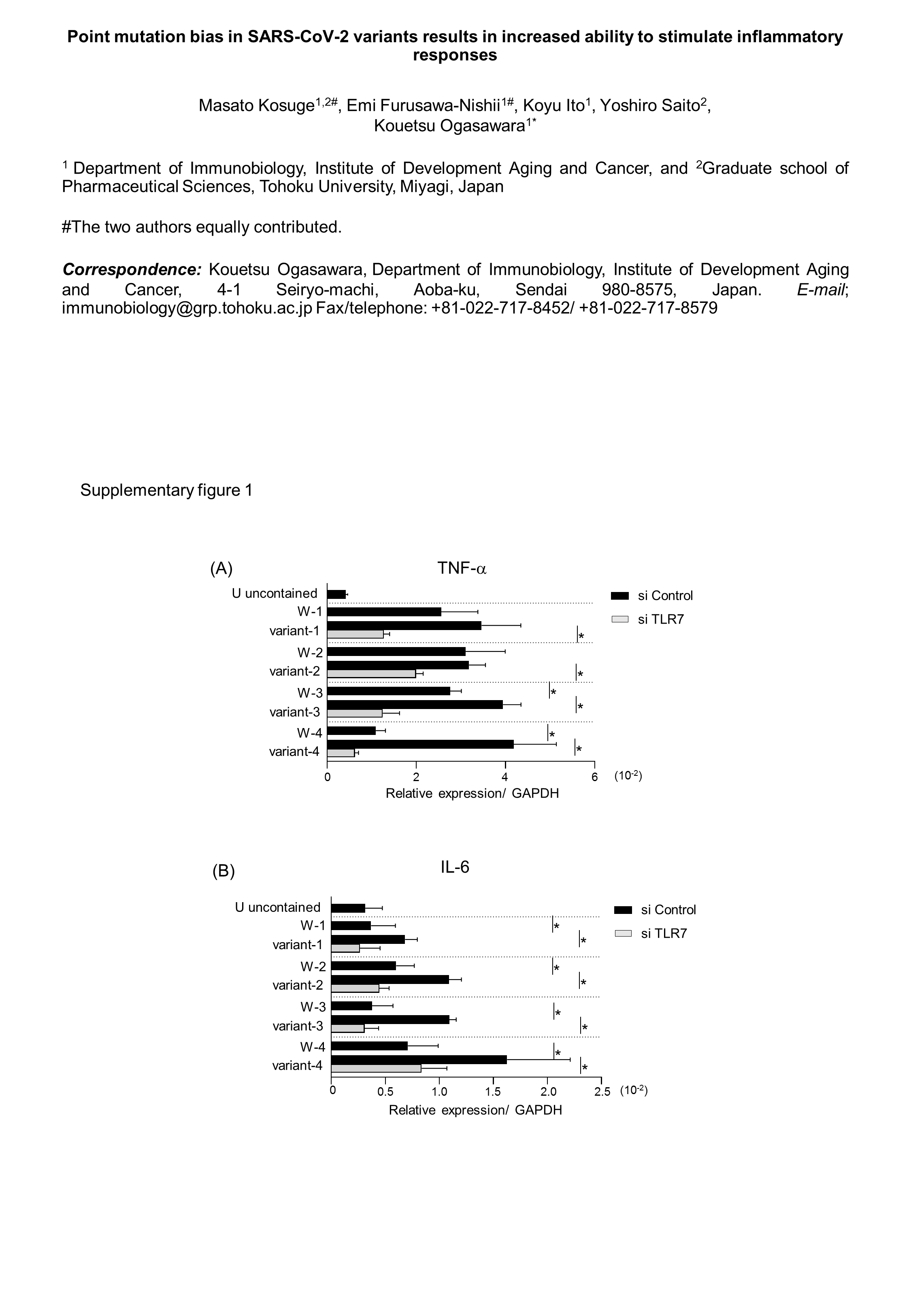

Supplement: Supplementary file 2 — Supplementary file2 [file 41598_2020_74843_MOESM2_ESM.tif]
